# Neuroimmunomodulation by estrogen in health and disease

**DOI:** 10.3934/Neuroscience.2020025

**Published:** 2020-10-30

**Authors:** Hannah P Priyanka, Rahul S Nair

**Affiliations:** Inspire Laboratory, Institute of Advanced Research in Health Sciences, Tamil Nadu Government Multi Super Speciality Hospital, Omandurar Government Estate, Chennai-600002, India

**Keywords:** neuroendocrine, norepinephrine, 17 β-estradiol, neural-immune, immunosenescence, reproductive aging, autoimmunity

## Abstract

Systemic homeostasis is maintained by the robust bidirectional regulation of the neuroendocrine-immune network by the active involvement of neural, endocrine and immune mediators. Throughout female reproductive life, gonadal hormones undergo cyclic variations and mediate concomitant modulations of the neuroendocrine-immune network. Dysregulation of the neuroendocrine-immune network occurs during aging as a cumulative effect of declining neural, endocrine and immune functions and loss of compensatory mechanisms including antioxidant enzymes, growth factors and co-factors. This leads to disruption of homeostasis and sets the stage for the development of female-specific age-associated diseases such as autoimmunity, osteoporosis, cardiovascular diseases and hormone-dependent cancers. Ovarian hormones especially estrogen, play a key role in the maintenance of health and homeostasis by modulating the nervous, endocrine and immune functions and thereby altering neuroendocrine-immune homeostasis. Immunologically estrogen's role in the modulation of Th1/Th2 immune functions and contributing to pro-inflammatory conditions and autoimmunity has been widely studied. Centrally, hypothalamic and pituitary hormones influence gonadal hormone secretion in murine models during onset of estrous cycles and are implicated in reproductive aging-associated acyclicity. Loss of estrogen affects neuronal plasticity and the ensuing decline in cognitive functions during reproductive aging in females implicates estrogen in the incidence and progression of neurodegenerative diseases. Peripherally, sympathetic noradrenergic (NA) innervations of lymphoid organs and the presence of both adrenergic (AR) and estrogen receptors (ER) on lymphocytes poise estrogen as a potent neuroimmunomodulator during health and disease. Cyclic variations in estrogen levels throughout reproductive life, perimenopausal surge in estrogen levels followed by its precipitous decline, concomitant with decline in central hypothalamic catecholaminergic activity, peripheral sympathetic NA innervation and associated immunosuppression present an interesting study to explore female-specific age-associated diseases in a new light.

## 1. Introduction

Homeostatic functions at the cellular and systemic level are maintained by cross-talk between the nervous system, endocrine system and immune system. This reciprocal communication among the homeostatic systems is essential for the maintenance of health, and any dysregulation in the communication leads to susceptibility and development of disease [1],[2]. Through several studies, it has been established that the hormonal secretion from the pituitary is tightly regulated by the hypothalamic neuroendocrine system through the release of hormones, neurotransmitters and neuropeptides; targeting endocrine glands to influence psychological and physiological functions including immunity [1],[3]. The immune system in turn also regulates systemic functions, through release of cytokines and chemokines that can cross the blood-brain barrier and thus, regulate the neural functions in the brain [4]. In addition to the indirect effects on immune functions, the nervous system can directly influence immune functions through synaptic associations of sympathetic NA and peptidergic nerve fibers by the release of norepinephrine (NE) and neuropeptides, respectively that innervate the primary and secondary lymphoid organs [5].

Aging is marked by decline in homeostatic maintenance in the neuroendocrine-immune network. Several studies have reported that age-associated dysregulation in the cross-talk between the neuroendocrine and immune system contributes to the development of diseases, in both rodents and humans [1],[2],[5],[6]. Aging in women, as compared to men, is different, evident by the distinct changes in the release of hormones and reproductive cyclicity, which in turn enhances the risk of development of female-specific diseases such as autoimmune disorders, hormone-dependent cancers, osteoporosis etc [7]–[9]. The crucial changes in hormonal secretion and reproductive cyclicity occur during middle age in both rodents and women, as they transition into acyclicity and menopause, respectively [3],[10],[11]. The menopausal transition is characterised by an early phase with delayed cycles and a late phase with more than 60 days of amenorrhea followed by a period of less frequent cycling and the final menstrual period spanning over a decade [12]. Estrogen levels surge during the early menopausal transition followed by a rapid decline spanning a period of 2 years before the final menstrual period is achieved and the levels stabilise again [12]–[14]. Thus the transition from regular cycles to menopause is a gradual process of hormonal changes that span multiple years, during which central and systemic functions are chronologically adapted to effect metabolic, immunological and neurological modulations.

## 2. Neuromodulation by estrogen

In females, reproductive aging is one of the first hallmarks of age-associated decline in the neuroendocrine-immune network. The depletion of ovarian follicles, hypothalamic deficits and the ensuing decline in the hypothalamo-pituitary-gonadal (HPG) axis and its mediators involving neurotransmitters, neuropeptides, releasing hormones [gonadotropin releasing hormone (GnRH) and luteinizing hormone (LH)] and target organs (ovaries) lead the transition into menopause [1],[3],[15]. This transition in females, begins during middle age before the onset of irregular cycles and a decline in GnRH-positive (GnRH^+^) neurons by a reduction in the ability of estrogen to induce neurochemical changes associated with pre-ovulatory surge in GnRH and LH [15]–[17]. Changes in the timing of the pre-ovulatory surges during middle age may be traced back to circadian rhythm disruptions due to deficits in the neurons of the hypothalamic suprachiasmatic nucleus [16]. The ability of estrogen to elicit GnRH/LH surge is attenuated much earlier than the actual reduction in GnRH^+^ neurons in the hypothalamus through decline in hypothalamic stimulatory molecules such as glutamate, NE, vasoactive intestinal peptide (VIP) and increase in inhibitory signals such as gamma-amino butyric acid (GABA) and opioids [3]. Proestrus spike in NE levels and tyrosine hydroxylase (TH) activity in the medial preoptic area and the arcuate nucleus were not observed in young and old dioestrus rats implicating estrogen-induced LH surge in central neurotransmitter release [17]–[21]. Thus centrally, estrogen plays a crucial role in the maintenance of neuroendocrine homeostasis in young females by inducing hypothalamic neurons and regulating the hormonal outflow through neurotransmitters and neuropeptides. The presence of estrogen seems vital in the maintenance of hypothalamic and hippocampal neurons that decline with reproductive age thereby highlighting the neuroprotective role of estrogen on central neurons by mediating antioxidant and antiapoptotic effects, modulating neurotrophic signals and promoting crosstalk. Peripherally however, the results are contradictory. The neuroprotective role of estrogen has been well established and yet, the perimenopausal estrogen surge in rodents has been shown to be implicated in the loss of sympathetic NA innervation of lymphoid organs leading to disruption in neuroendocrine-immune homeostasis in the periphery [22],[23]. Treatment of ovariectomised middle-aged Sprague-Dawley (SD) rats with estrogen [0.6 µg and 300 µg timed-sustained release (SR) pellets/30 days] significantly decreased the expression of p-TH in the mesenteric lymph nodes [24]. The onset of menopause is characterised by a dramatic decline in circulating levels of estrogen leading to a hypoestrogenic state that have lasting implications on the reproductive and non-reproductive functions of the hormone ranging from loss of rhythmicity in neurotransmitter dynamics in the brain to a decline in peripheral immune responses [25],[26]. Thus in the periphery, the estrogen surge in the perimenopausal/early transition phase is implicated in the loss of sympathetic nerve fibers in the lymphoid organs leading to dysregulation of neural-immune homeostasis. The effects of estrogen can perhaps be understood from a dose-response perspective. The higher circulating levels during menopausal transition in the periphery may be neurodegenerative although centrally, estrogen seems to exert a neuroprotective role.

## 3. Immunomodulation by estrogen

In women, regular menstrual cycles in the young give way to irregular cycles by middle-age, leading to perimenopausal estrogen surge, onset of menopause leading to the final menstrual period. Rhythmic cyclicity of ovarian hormones including estrogen are implicated in the alteration of cell-mediated immune responses in follicular and luteal phases of the menstrual cycle. Cell-mediated immune functions including mitogen-induced proliferation and cytokine production were significantly enhanced during follicular phase of the menstrual cycle in young women compared to the luteal phase [25]. In the lymphoid organs, estrogen is involved in the regulation of maturation, differentiation, activation and proliferation of lymphoid cells thereby influencing immune effector molecules like cytokines and antibodies [27]. Estrogen modulates Th1/Th2 cytokine balance in favour of Th2 type and influences humoral responses to diseases [28]–[29]. In murine models, estrus cycle-associated fluctuations in the levels of circulating gonadal hormones have been shown to alter T and B cell proliferation, and localization of immunoglobulin-A (IgA)-producing plasma cells [30]. Immunomodulation by estrogen is concentration dependent and varies according to the nature of the immune stimulus, the receptor subtype profile of the target organ, and the timing of administration [31]–[34]. Preovulatory surge in estrogen inhibits the translocation of nuclear factor-kappa B (NF-kB) through transcriptional repression of Ikbkg and affect the maturation of dendritic cells in the lymph nodes [32]. On the other hand, low dose estrogen treatment of ovariectomised rats enhanced interferon-gamma (IFN-γ) production and antigen-specific T cells [33],[34]. Thus it is implicated in the predominant incidence of autoimmune diseases, hormone-dependent cancer, osteoporosis, and cardiovascular diseases through alterations in T cell functions and cytokines during reproductive aging in females [35]–[37]. Centrally, estrogen mobilises astrocytes and microglia and plays a neuroprotective and anti-inflammatory role through neurotrophin-mediated growth, survival and neuronal plasticity [38]–[40]. Hypothalamic neuronal deficits and peripheral sympathetic denervation share a cause-effect relationship with age-associated decline in estrogen and associated immunosuppression [9],[18],[19],[22]. Thus estrogen plays a clearly defined role in influencing the immune responses in women depending upon the phase of menstrual cycle, reproductive age, levels of circulating estrogen, and influences the type of response elicited by modulating the Th1/2 balance.

## 4. Estrogen-mediated reversal of adrenergic immunosuppression

The presence of sympathetic NA nerve fibres in synaptic association with immune organs, and the expression of adrenergic and steroid hormone receptors on immune cells renders immune cells susceptible to the immunomodulatory effects of both neurotransmitters and steroid hormones alike. Of interest here is the interesting crosstalk between NE signals released from the sympathetic fibres and circulating estrogen, both of which have shown contradictory effects on immune cells.

The expression of adrenergic receptor (AR) subtypes including α1-, α2-, β1-, β2-adrenoceptors have been characterised in several immune cell subsets including splenic lymphocytes (T cells and B cells) and natural killer (NK) cells [2]. Published data from our laboratory has shown that predominantly, adrenergic stimulation of splenic lymphocytes is immunosuppressive. Treatment of splenocytes with α1-AR agonist phenylephrine, significantly decreased splenocyte IFN-γ expression (10^−3^ M, 10^−6^ M and 10^−9^ M) while α2-AR agonist clonidine (10^−6^ M and 10^−9^ M) inhibited splenocyte proliferation, phosphorylated-extracellular signal regulated kinase (p-ERK) and phosphorylated-cAMP response element binding protein (p-CREB) expression [41]. β2-AR agonist terbutaline (10^−6^ M and 10^−9^ M) significantly decreased splenocyte proliferation and interleukin-6 (IL-6) expression in splenic lymphocytes [42]. Estrogen co-treatment significantly reverses immunosuppression by α1-, α2-, and β2-adrenoceptor agonists. Co-treatment of splenocytes with 10^−9^ M 17 β-estradiol reverses phenylephrine-mediated decline in IFN-γ expression, clonidine-mediated decline in proliferation, p-ERK and p-CREB expression and terbutaline-mediated decline in proliferation and IL-6 expression [41],[42]. 1-ARs are expressed on neutrophils, mast cells, macrophages, dendritic cells and microglia where they are known to mediate inhibition of neutrophil functions, mast cell histamine release, macrophage tumor necrosis factor-alpha (TNF-α) expression, dendritic cell migration and suppression of microglial activation [43]–[47]. Unpublished data from our laboratory has shown that coincubation of splenic lymphocytes with 1-AR agonist dobutamine, suppresses splenocyte proliferation thereby exerting a similar immunosuppressive effect. These studies have shown that sympathetic modulation of immune cell functions may be crucial for the maintenance of naïve cells in the lymphoid organs. Exposure to estrogen overrides the immunosuppressive role of NE and stimulates immune functions. Reproductive aging is associated with concomitant loss of sympathetic NA fibers and associated naive cells possibly due to compounded effect of prolonged exposure to circulatory estrogen throughout reproductive life and estrogen surge during early menopausal transition.

## 5. Mechanistic role of ERs

Intracellular (genomic and non-genomic) effects of estrogen are mediated by specific intracellular and membrane receptors namely ERα and ERβ. ERα is predominantly expressed on CD4^+^ T cells while ERβ predominantly expressed on B cells, playing a role in maturation and differentiation of thymic T cells and B lymphopoiesis in the bone marrow [48]. Once estrogen binds to these receptors, they control the expression of their target genes by binding to the estrogen response element (ERE) leading to the activation of several downstream signals including NF-κB, activator protein-1 (AP1) or ERK [48]–[49]. Studies from our laboratory have shown that, the effects of estrogen on cell mediated immune functions are concentration dependent and are differentially mediated through specific receptor subtypes through antioxidant enzymes and nitric oxide (NO) [34]. Thus the effects of estrogen on cell-mediated immune functions may be dependent upon the type of immune cells and mediated through ERα and ERβ [50],[51]. ERα mediates the expression of Th1 cytokines and proliferation of CD4^+^ T cells in normal rodents and experimental autoimmune disease model of myasthenia gravis [33],[52]. ERα-mediated suppression of NF-κB through regulation of let-7a and miR-125b microRNAs have been shown to play a role in estrogen-mediated suppression of inflammatory process in primary macrophages [53],[54]. Expression of ERα-mediated signals in the splenocytes has been implicated in the suppression of inflammation in trauma-haemorrhage through IL-6 and TNF-α [55]. These studies have shown that ERα plays a predominantly anti-inflammatory role favouring Th1 responses and suppressing inflammatory signals. The involvement of both ERα/β have been shown in protection against bacterial infection and sepsis in an animal model of trauma-haemorrhage by reversing the suppression of T cell proliferation and inducing Th1 and Th2 cytokine production [56],[57]. In mouse models of cardiotoxin-induced myoinjury, estrogen exerts anti-inflammatory effects through infiltration of anti-inflammatory M2-macrophages and T-regulatory cells and suppressing Th1 responses through ERβ [58]. Estrogen can exert its effects across immune cell subsets through receptor-dependent and receptor independent mechanisms and mediate specific effects [59].

Crosstalk between estrogen and adrenergic signalling has also been implicated in the expression of proinflammatory cytokines by peritoneal macrophages [60]. In an *in vitro* study conducted in our laboratory, estrogen [10^−6^ M] treatment of splenic lymphocytes reversed immunosuppression mediated by adrenergic agonists and enhanced cell-mediated immune functions [41],[42]. Apart from ERs, estrogen can also bind to membrane-bound G protein-coupled estrogen receptor (GPER) and regulate T cell mediated immune functions [61]. ER-mediated activation of ERK/mitogen activated protein kinase (MAPK), CREB, and Akt pathways have been shown in MCF-cells, immortalised neuronal cultures, Parkinson's disease and glioma models [62]–[65]. Estrogen has been shown to modulate the tumor-immune microenvironment in female mouse models of liver cancer through ERα and ERβ [66].

Centrally, the effects of estrogen are transduced through the activation of ERK and CREB in the hypothalamus and hippocampus, activation of Akt signalling pathways in the striatum or mediating anti-inflammatory effects through inhibition of NF-κB [67]–[73]. Estrogen augments TH promoter activity through specific pathways involving membrane-bound ERα in PC-12 neuronal cells and ERβ in mouse locus coeruleus [74]–[76]. The expression of ERβ and ERα in the adult female brain is significantly altered with reproductive age and estrogen supplementation in specific brain areas and their relative expression may modulate downstream signalling responses in order to influence memory, cognitive functions, neuronal plasticity, inflammation and neurodegeneration [77],[78]. Evidence from estrogen replacement therapy studies has shown that the synthetic steroid, Tibolone with weak estrogenic, progestogenic and androgenic activity decreased body weight, glucose tolerance, triglycerides and cholesterol levels in SD rats fed with high-fat high-fructose diet compared to standard diet by modulating the expression of ERs in the brain area [79]. Thus estrogen can influence neuroimmunomodulation both centrally and peripherally through specific intracellular pathways based on the dose, type of receptor subtype activated and presence of co-stimulatory/inhibitory signals.

## 6. Estrogen and neuroprotection

The role of 17 β-estradiol in neuroprotection in young and old females has been widely explored in a number of studies. We have shown that estrogen exerts a dual role by mediating neuroprotective effects in specific brain areas while causing sympathetic denervation in the periphery esp. lymphoid organs [22],[25]. Administration of a single dose of 17 β-estradiol (10 pg/kg s.c.) or using SR pellets (0.1 pg/pellet/3 weeks), to ovariectomised adult virgin female SD rats similar to proestrus surge in estrogen have both shown significantly decreased sympathetic innervation in the uterine smooth muscles [80]. Similarly, ovariectomised adult female Long Evans hooded rats treated with estrogen dose of 40–55 µg/kg/d orally up to 8 months showed increased TH-positive (TH^+^) nerve fibers in the medial prefrontal cortex of old rats [81]. In our study, ovariectomised middle-aged female SD rats supplemented with estrogen through SR pellets implanted at the scruff (300 µg) for 30 days showed increased splenic p-TH and nerve growth factor (NGF) expression [82]. However, estrogen (0.6 µg) supplementation of ovariectomised middle-aged SD rats enhanced TH expression in frontal cortex and hippocampus, suppressed NF-κB expression in the frontal cortex and striatum, and decreased NGF expression in the hippocampus [83],[84]. Long-term effects of estrogen exposure in the periphery are implicated in the early onset of peripheral sympathetic denervation of lymphoid organs in females compared to males [22]. Age-associated decline in estrogen is concomitant with the development of cognitive impairment and neurodegenerative diseases through decline in basal forebrain neurons, inhibition of NO and cholinergic functions, loss of trophic factors and onset of neuroinflammation [72],[73],[83]–[89]. Estrogen plays a neurotrophic role promoting plasticity in hippocampal neurons through expression of growth factors, signalling molecules, cholinesterases and boosting metabolism [73],[90]–[91]. Estrogen-induced synthesis of catecholamines through modulation of TH^+^ fiber density and TH activity in the hippocampus and frontal cortex have been implicated in memory and cognitive functions [73],[92]–[95]. The trophic effects of estrogen begins early in the brain development showing neuroprotective effect that may modulate neurodevelopment of different brain structures as evident with studies reporting differences in men and women with movement disorders [96]. Central neuroprotective effects of estrogen clearly substantiate the claim that estrogen is beneficial for the brain. However, neuroprotection by estrogen cannot be extrapolated to the periphery which is exposed to fluctuating levels of estrogen throughout reproductive life and during menopausal transition.

## 7. Estrogen and the antioxidant machinery

Aging takes a toll on cellular processes through increased allostatic load of reactive oxygen species and decline in antioxidant machinery leading to impaired functions and cellular distress. Peroxides and superoxide anions can bind to cell membrane lipids through malondialdehyde adducts leading to impaired structural integrity that eventually compromise functional integrity of the cell [97],[98]. Collectively, these changes affect neuroendocrine-homeostasis leading to dysregulation and development of age-associated diseases. Studies using splenic lymphocytes from old rats have shown age-associated increase in lipid peroxidation, accumulation of protein carbonyl compounds leading to enzyme dysfunctions, disruption of cellular functions and immunosenescence [73],[81],[99]. Estrogen (10^−12^ M, 10^−10^ M and 10^−8^ M) plays a potent antioxidant function in splenic lymphocytes by increasing the activities of superoxide dismutase (SOD), catalase (CAT) and glutathione peroxidase (GPx) *in vitro* [34]. Ovariectomised female rats showed significantly reduced chemotaxis index, lymphocyte proliferation and NK cell activity compared to their age-matched and young controls [82],[100]–[102]. In ovariectomised animals supplemented with estrogen through SR pellets (0.6 and 300 µg) for 30 days, there was a significant reversal of age-associated decline in antioxidant enzyme activities and accumulation of reactive oxygen species, lipid peroxides and protein carbonyls in lymphoid organs and brain areas [73],[82]. Physiological levels of estrogen may promote female longevity through the upregulation of antioxidant signals through survival signalling (MAPK, ERK) or anti-inflammatory signalling (NF-κB) cascades both centrally and peripherally [25],[103]. Further, estrogen confers protection against mitochondrial stress, and ERα has been linked to mitochondrial unfolded protein response [UPR (mt)], that orchestrates several pathways, including antioxidant machinery [104]. In the brain, estrogen-mediated neuroprotective effects in healthy aging, cerebral ischemia and Alzheimer's disease also involve its potent ability to suppress cellular oxidative stress by enhancing antioxidant activities in various brain regions [68],[105]–[106]. Antioxidant effects of estrogen play a crucial role in neuroimmunomodulation in the periphery thereby enhancing immune cell functions and neuronal integrity, suppressing inflammation and maintaining neural-immune homeostasis in the young and in healthy aging.

## 8. Estrogen and energy metabolism

The brain is a glucose-intensive organ and hence places a huge physiological demand for energy currency on the systemic metabolic process [107]. Estrogen plays a crucial role in meeting this demand by upregulating the expression of glucose transporters to facilitate glucose transport across the blood brain barrier, enhancing the glycolytic process by influencing p53, glycolytic enzymes and regulation of the tricarboxylic acid (TCA) cycle and enhancing mitochondrial integrity [108]–[112]. Estrogen can modulate the expression and activity of specific isozymes of glycolytic enzymes such as hexokinase, phosphofructokinase and pyruvate kinase in rat neuronal cells [113],[114]. Hormone replacement studies in women undergoing perimenopause, early menopause and surgical menopause have shown that estrogen enhances glucose metabolism in the brain, attenuates neuroinflammation and prevents cognitive decline [115]–[119].

Another target of estrogen is the Na^+^/K^+^/ATPase pump that is energy intensive and is crucial for the maintenance of ionic gradients, resting potentials and triggering action potentials that is also regulated by catecholamines mainly NE and serotonin (5-HT) [110],[111]. Estrogen treatment of ovariectomised female SD rats enhances the activity of Na^+^/K^+^/ATPase activity in the frontal cortex and hypothalamus conferring neuroprotection against amyloid-β-induced oxidative stress and suppressing lipid peroxidation [120],[121]. During aging and in age-associated degenerative diseases such as Alzheimer's and Parkinson's, there is a decline in mitochondrial regulation of oxidative phosphorylation primarily due to cytochrome C oxidase leading to an energy-defunct state that aids in the progressive degeneration [112],[122],[123]. Estrogen treatment enhanced the activity of cytochrome C oxidase in the frontal cortex, medial basal hypothalamus and hippocampus and promoted neuronal activity [124]. Thus the regulators of energy metabolism, mitochondrial integrity and transporters by estrogen plays a crucial role in the etiology and pathology of these diseases [125],[126].

## 9. Conclusions

**Figure 1.**
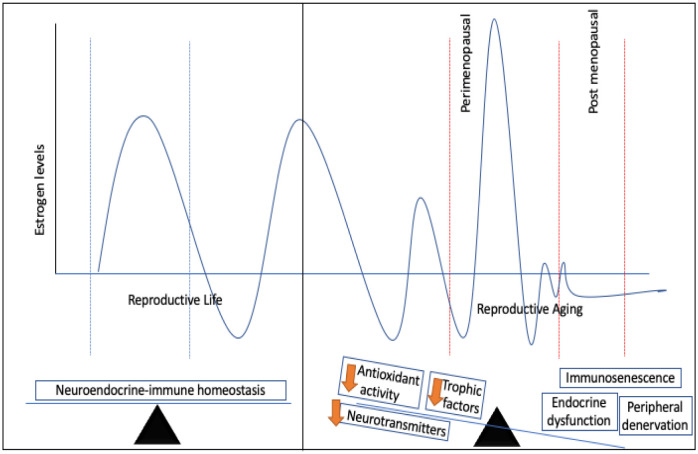
Estrogen levels rhythmically fluctuate throughout reproductive life. In young healthy females, the robustness of the neuroendocrine-immune network maintains physiological homeostasis by mutual regulation between the neural, endocrine and immune mediators. During reproductive aging however, perimenopausal surge in estrogen followed by its precipitous decline post menopause leads to neuroendocrine-dysfunction, peripheral denervation and immunosenescence compounded by the lack of trophic factors, neurotransmitters, signalling molecules and defunct antioxidant machinery.

The diverse effects of estrogen on the neuroendocrine network and the dependence of the female physiology on the robustness of estrogen-associated regulatory functions during reproductive life and aging places women at risk of developing autoimmune diseases, heart diseases, osteoporosis, neurodegenerative diseases, and breast cancer. While loss of neuroendocrine-immune homeostasis shares a cause-effect relationship with impaired hormone secretion, immunosenescence, peripheral denervation and central neurodegeneration, decline in neurotransmitters etc, the cumulative effects set the stage for age-associated disease pathology (Figure 1). Age-associated disruption of neuroendocrine-immune homeostasis is exacerbated by dysfunctional antioxidant machinery, increased allostatic load, decline in biosynthesis of trophic factors, signal transduction molecules and poor energy metabolism. Although loss of estrogen precludes the deregulation of neuroendocrine-immune homeostasis in females, the answer to restoring homeostasis isn't as simple as estrogen replacement. This is due to the effects of estrogen as a double edged sword beneficial for neuroprotection in the central nervous system while mediating peripheral denervation in the lymphoid organs and enhancing the risk of cardiovascular diseases and breast cancer. Estrogen therefore is pivotal to the maintenance of homeostatic integrity in females and its effects are largely dependent upon the dose, time of secretion, nature of receptors and target organ. Mechanistic effects of estrogen may result in the transcription of genes regulated by specific response elements and contribute to a plethora of functions including cell-mediated immunity, Th1/Th2 cytokine balance, neuroprotection, growth factor biosynthesis, NE release, feed-back regulation of HPG axis, peripheral denervation, all of which may fuel the loop towards regulation or dysregulation in health and disease.
